# Dietary Antioxidant Capacity Promotes a Protective Effect against Exacerbated Oxidative Stress in Women Undergoing Adjuvant Treatment for Breast Cancer in a Prospective Study

**DOI:** 10.3390/nu13124324

**Published:** 2021-11-30

**Authors:** Luiza Kuhnen Reitz, Jaqueline Schroeder, Giana Zarbato Longo, Brunna Cristina Bremer Boaventura, Patricia Faria Di Pietro

**Affiliations:** 1 Post Graduate Program in Nutrition, Federal University of Santa Catarina, Florianópolis 88040-900, Brazil; luizakreitz@gmail.com (L.K.R.); jaqueline.schroeder04@gmail.com (J.S.); giana.zarbato@gmail.com (G.Z.L.); 2 Nutrition Department, Federal University of Santa Catarina, Florianópolis 88040-900, Brazil; brunnab@gmail.com

**Keywords:** dietary antioxidant content, antioxidant intake, breast neoplasm, oxidative stress, adjuvant chemotherapy, adjuvant radiotherapy

## Abstract

Breast cancer (Bca) is the most common type of cancer among women worldwide, and oxidative stress caused by adjuvant treatment may be decreased by antioxidant intake. The aim of this study is to investigate the associations between Dietary antioxidant Capacity (DaC) and oxidation and antioxidant biomarkers in women undergoing adjuvant treatment (AT) for Bca. This prospective study had a sample of 70 women (52.2 ± 10.7 y). DaC (mmol/g) was calculated using nutritional data obtained from a Food Frequency Questionnaire, and blood was collected to measure the oxidation and antioxidant biomarkers at baseline (T0), and after AT (T1). Carbonylated protein levels were inversely associated with DaC at T1 (*p* = 0.004); women showed an increased risk of having increment on lipid hydroperoxides and thiobarbituric acid reactive substances (TBARS), and decrement on ferric reducing antioxidant power (FRAP) and reduced glutathione after AT, in response to lowered DaC (*p* < 0.05). Carbonylated proteins, TBARS and FRAP levels remained stable between the periods for women at the 3rd DaC tertile at T1, differentiating them from those at the 1st tertile, who showed negative changes in these biomarkers (*p* < 0.04). DaC may be beneficial for women undergoing AT for Bca, since it promoted a reduction in oxidative stress.

## 1. Introduction

Female breast cancer is the most diagnosed cancer, with an estimated 2.3 million new cases (11.7%) and it is the leading cause of cancer-related death among women worldwide [1]. In Brazil, 66,280 new cases of breast cancer are expected for each year of the 2020–2022 period, corresponding to an estimated risk of 61.61 new cases in every 100,000 women [2]. Recent investigations have shown that breast cancer is a heterogeneous group of diseases, and the main difference between them is the prognosis, and the different responses to treatment [3,4].

Diet and lifestyle are frequently pointed to as risk factors for breast cancer [5,6,7] since they are strongly related to the production of Reactive Oxygen Species (ROS). The existing link between nonmodifiable risk factors and oxidative stress reinforces the evidence of the influence of ROS in the initiation, promotion and progression of breast cancer [8]. Higher ROS levels promote sustained cell proliferation and survival, inflammation, angiogenesis and metastasis and, in this sense, oxidative stress is well established as a carcinogenic factor [9,10]. Controversially, chemotherapeutic and radiotherapeutic agents may cause injury to DNA, leading to malignant cell death via ROS generation. The oxidative stress-related mechanisms of malignant cell death are also related to the side effects of these treatments, such as gastrointestinal disturbances (nausea, vomiting and food aversions), and other deleterious effects (cardiac damage, pulmonary complications, kidney toxicity, and fertility problems) [11].

Antioxidants are compounds that inhibit the oxidation of other molecules by neutralizing ROS and other reactive species [12], which can be enzymatic, such as superoxide dismutase or catalase, or nutrient-delivered molecules, consumed with foods or by dietary supplement intake [13,14,15,16]. Research has shown that oral antioxidant supplementation may reduce side effects of chemo and radiotherapy, leading to better quality of life [11,15]. However, there is also a controversy around the positive effect of antioxidant supplementation on cancer treatment, since it was associated with lower therapeutic efficacy, and cancer recurrence [17,18,19].

Previous studies have shown that a higher content of dietary antioxidants, obtained through food, was associated with lower oxidative stress biomarkers in healthy adults [20,21]. In this context, the Dietary antioxidant Capacity (DaC) is a useful tool for evaluating the dietary antioxidant content, since it considers all type of food antioxidants present in the diet, and the synergistic effects between them [22,23]. It has been shown that DaC is inversely associated with breast cancer risk [24,25,26], but there is lack of evidence regarding the influence of DaC on oxidative stress in women undergoing adjuvant treatment for breast cancer. 

Considering the emerging burden of breast cancer on public health worldwide, and the perspective of the lower efficacy of cancer treatment in response to antioxidant supplementation, it is important to identify the impact of dietary antioxidant intake on oxidative stress biomarkers. It is noteworthy that oxidative stress is necessary for malignant cell death, but is also highly involved in adverse effects of adjuvant treatment on healthy cells, and in disease recurrence, which can have a strong impact on breast cancer prognosis. 

The aim of the present study is to investigate the effect of DaC on oxidative stress in women undergoing adjuvant treatment. We hypothesized that a higher content of antioxidants in the diet may protect women with breast cancer from exacerbated oxidative stress provoked by adjuvant treatment.

## 2. Materials and Methods

### 2.1. Study Design, Sampling and Ethics

**Figure 1:**
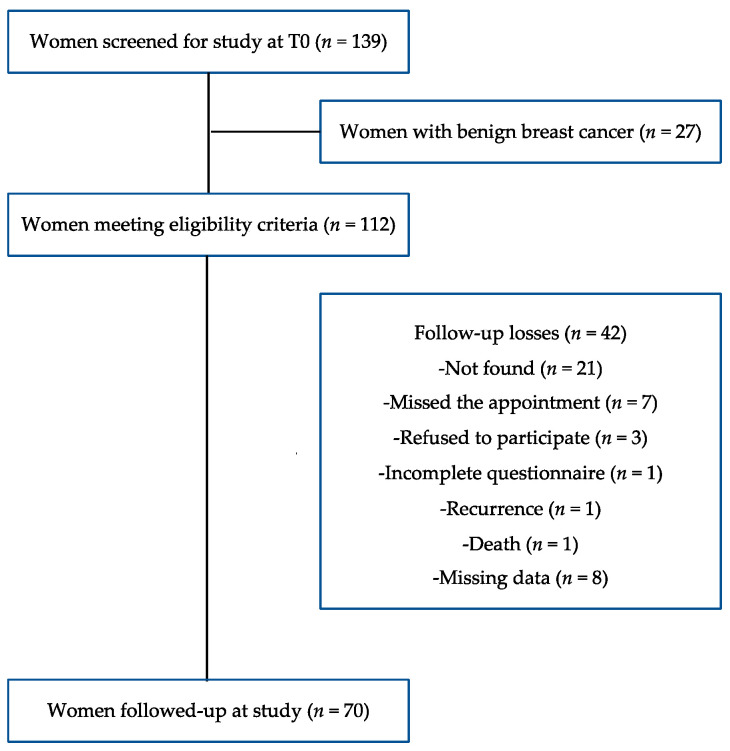
Flowchart of participant selection at baseline and follow-up.

This is a prospective study performed with a convenience sample composed by all women admitted for surgical treatment for breast cancer at Carmela Dutra Maternity between 2006 and 2011. The sample included women with confirmed malignancy by anatomopathological report issued after surgery, who underwent adjuvant treatment at Cancer Research Center (CEPON) in Florianopolis/SC, Brazil. The exclusion criteria were: women without confirmed malignancy; previous history of cancer or surgery in a period shorter than one year; women who had undergone neoadjuvant treatment; pregnancy or lactation; women positive for human immunodeficiency virus [27]. From the 139 women recruited at baseline, there were 70 with a full set of data who signed the written consent who were included in this study, after some losses (Figure 1). 

Data were collected at baseline (T0) and after adjuvant treatment (T1), with an average of 14 ± 4.4 months between the periods. The effect size for the primary outcomes (oxidative stress biomarkers) was calculated using retrospective power (1 − β) and where *n* = 70. The calculation was performed in the G*Power software^®^ (Heinrich-Heine-Universitat Dusseldorf, Dusseldorf, Germany), which resulted in a power of 83%.

The present study was approved on 30 May 2008 by the Human Research Ethics Committee of Federal University of Santa Catarina, by the Ethics Committee of Carmela Dutra Hospital, and by the Cancer Research Center (CEPON) Ethics Committee (process number 099/08), and was conducted in accordance with the Declaration of Helsinki.

### 2.2. Sociodemographic, Anthropometric and Clinical Data

An interview to collect sociodemographic, anthropometric and clinical data was performed by trained interviewers. The data were collected using a questionnaire developed by Di Pietro et al. [28] and adapted by Rockenbach [29] at T0 and T1, which contains issues about identification, sociodemographic data, familiar cancer history, reproductive and clinical history in relation to cancer, lifestyle questions (physical activity, smoking, alcohol intake), and anthropometric information. The staging of breast cancer was classified by a mastologist according to the TNM system [30]. Additionally, a “clinical-nutritional questionnaire after treatment” was used at the T1 stage to obtain the following information: type of antineoplastic treatment performed, drugs received, food aversions developed in response to adjuvant treatments, side effects of adjuvant treatments, and nutritional supplementation use after diagnosis [29]. 

Weight (kg) was measured using a digital scale (Marte Científica e Instrumentação Industrial LTDA^®^, Santa Rita do Sapucaí, Brazil; model PP, 180 kg capacity and 50 g precision), and height (m) by a stadiometer (1 mm precision), to calculate Body Mass Index (BMI), which was classified according to World Health Organization (WHO) cut-off points [31]. Using an inelastic anthropometric tape with an accuracy of 0.1 cm, waist circumference was measured, and classified by WHO criteria [32]. Patients reported the frequency and duration of physical activity performed weekly, and this was used to calculate the Physical Activity Level (PAL), which is the ratio between the total estimated energy expenditure and basal energy expenditure, which was classified according to Institute of Medicine [33]. 

### 2.3. Dietary Antioxidant Capacity Assessment 

Food intake was assessed by a trained interviewer using a Food Frequency Questionnaire (FFQ) [34,35] at T0 and T1. The FFQ was related to the usual food intake from the previous year, and so, the food consumption corresponds to the year before the diagnosis and the year that adjuvant treatment occurred, for T0 and T1, respectively. For seasonal foods, the harvest period was considered [36], and a calculation was carried out to convert the reported intake during harvest period into daily consumption. Food intake from FFQ was converted in daily quantitative information to calculate the DaC and total energy consumed [37,38,39]. 

DaC was calculated from daily quantitative information of food intake obtained with FFQ, using a database of antioxidant power based on the Ferric Reducing Antioxidant Power (FRAP) method, based on 3139 foods from Scandinavia, USA, and, from Europe, South America, Africa and Asia [40]. Data about FRAP concentrations on foods were transformed in mmol/g for calculation purposes, since they are originally expressed in mmol/100 g. When a food usually consumed was not available on the database, the antioxidant power from foods with similar nutritional composition was used, regarding minerals, vitamins, and bioactive compounds with antioxidant action [38,39,41,42,43], or foods from the same botanical group. For foods for which the cooking method was not described on the FFQ, the figure used for antioxidant power was the average of the raw and cooked, values, when they were available on the database. For food with more than one option on the database, an average value was used for antioxidant power. After calculating each food, DaC is the sum of the antioxidant power from all foods in habitual diet and the synergistic effect between them [22,23]. For analysis purposes, plant foods were grouped considering the Food Guide for Brazilian Population [44] as follows: whole cereals, legumes, tubers and roots, total fruits, and total vegetables. Regarding the main type of antioxidant, they were grouped as follows: cruciferous vegetables, rich in isothiocyanates [45]; orange and dark green vegetables and fruits, rich in beta-carotene [38]; citric foods, rich in Vitamin C [38]; red vegetables and fruits, rich in lycopene [46]; and polyphenol-rich foods and beverages, rich in polyphenols [47,48].

### 2.4. Oxidative Stress Biomarkers Analyses

#### 2.4.1. Blood Collection

**Figure 2:**
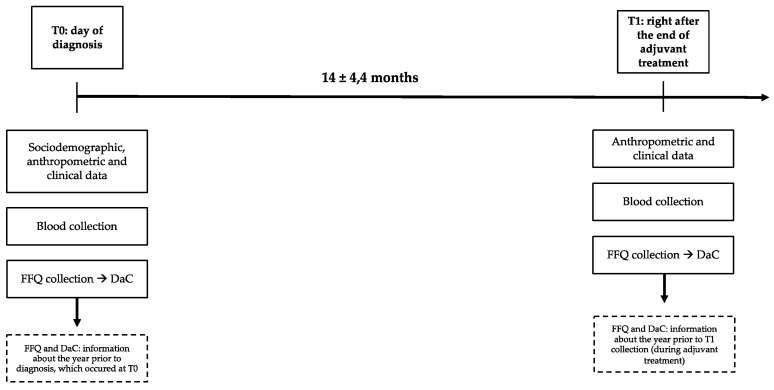
Timeline of the data collection.

A venipuncture in the forearm was performed at T0 and T1, without fasting, to collect 15 mL of blood into two tubes with and without EDTA, to obtain plasma and serum, respectively, by centrifugation (1000× *g*/10 min). The blood and FFQ were collected on the same day (at T0 and T1); T0 was the day of diagnosis and T1 was a moment right after adjuvant treatment. FFQ and therefore DaC at T0 corresponds to dietary antioxidant power at the year prior to diagnosis, considering the retrospective characteristic of the FFQ; DaC of T1 corresponds to dietary antioxidant power at the year in which adjuvant treatment occurred (Figure 2). 

#### 2.4.2. Antioxidant Biomarkers Analyses

Before the centrifugation, an aliquot from the whole blood was separated to perform the measurements of concentrations of reduced glutathione (GSH), using 20% trichloroacetic acid for protein precipitation, and 2-nitrobenzoic acid (DTNB) for color development, as described by Beutler et al. [49]. The serum antioxidant Capacity was analyzed with the Ferric Reducing Antioxidant Power (FRAP), according to method described by Benzie and Strain [50], based on the reduction of Fe^3+^ on Fe^2+^ chelated by 2,4,6-Tri(2-pyridyl)-s-triazine (TPTZ) for absorbance reading. 

#### 2.4.3. Oxidation Biomarkers Analyses

Lipid peroxidation was measured using the concentrations of lipid hydroperoxides (LH) and substances reactive to thiobarbituric acid (TBARS) in plasma. LH concentrations were obtained by the oxidation of iron with xylenol orange, based on the rapid oxidation of Fe^2+^ on Fe^3+^ in acid medium, mediated by the LH present in the sample [51]. The TBARS assay was based on the reaction of one molecule of malondialdehyde (MDA) with two molecules of thiobarbituric acid, resulting in two H_2_O molecules and a pink pigment [52]. Carbonylated protein concentrations were measured to identify the plasmatic protein oxidation level, based on the reaction between free radicals and protein residuals originating from products in the carbonyl group, which can be measured by their reaction with 2,4-Dinitrophenylhydrazine [53].

TBARS, LH, GSH and FRAP assays were performed on the same day as blood collection, and the carbonylated protein assay was performed after storage of the plasma sample at −80 °C for a maximum of 30 days. All oxidative stress and antioxidant biomarkers were analyzed in duplicate, and expressed as μmol/L. 

### 2.5. Statistical Analysis

Data were entered in Microsoft Office Excel^®^, (Microsoft Corporation, Washington, DC, USA) and subsequently imported to STATA^®^ software version 14.0 (STATA corp LLC, College Station, United States of America) to carry out statistical analysis. All variables were tested for normality of distribution by Shapiro–Wilk and skewness tests, and were reported as mean ± standard deviation (SD), and median and interquartile range for symmetric and asymmetric distribution, respectively. The comparison of characteristics between DaC tertiles at T0 and T1 was tested using ANOVA or the Kruskal–Wallis test for continuous variables with symmetric and asymmetric distributions, respectively, and the Chi-square test for categorical variables; the latter expressed as absolute and relative (%) value. The differences in continuous variables between T0 and T1 were analyzed by paired *t*-test or Wilcoxon test, depending on normality of data. 

DaC was adjusted for energy intake by the residual method [54]. TBARS, LH and carbonylated proteins were logarithm-transformed to be included in multiple linear models, which were used to investigate the association between energy-adjusted DaC, oxidation and antioxidant biomarkers. To evaluate whether the reduction in energy-adjusted DaC promotes an increase in oxidative biomarkers and decrease in antioxidant biomarkers, models of logistic regression were applied by dichotomizing the dependent and the main independent variables. The adjusted models of linear and logistic regressions considered the following confounder variables: tumor stage, tumor type, lymph node involvement, race, schooling, alcohol intake, smoking status, nutritional supplement use, BMI, WC, and PAL at T0. At T1, variables in adjusted models were also included: type of mammary surgery, type of treatment, type of hormone therapy, number of chemotherapeutic and radiotherapeutic sessions. In all final adjusted models, only confounding variables presenting the regression coefficient values at *p* ≤ 0.20 were included, except for age, which was included in all adjusted models. Statistical significance level was set at 5%.

## 3. Results

The sample was composed of 70 women aged 52.2 (±10.7) years), who were submitted to adjuvant treatment over 6.9 (±4.7) months (radiotherapy, chemotherapy or combination). Regarding the general characteristics of the sample (Table 1), women with higher dietary antioxidant content were more likely to be younger and to show lower BMI and WC, compared to women who exhibited a lower consumption of dietary antioxidants (*p* < 0.05).

DaC values did not differ between T0 and T1 (Table S1). The food group that most contributed to DaC was the polyphenol-rich food and beverages, followed by total fruits, and a decrease in intake of antioxidants from these food groups after adjuvant treatment was observed. The main contributor to DaC was coffee (T0 = 58.5% and T1 = 45.2% of DaC). Regarding oxidation and antioxidant biomarkers, women showed increased levels of TBARS, lipid hydroperoxides, carbonylated proteins, and lowered levels of FRAP after adjuvant treatment (*p* ≤ 0.05). No differences in oxidation and antioxidant biomarkers between the tertiles of DaC at T0 and T1 were observed (Table S2).

Table 2 shows that women at the lowest tertile for DaC at T0 were more likely to have increased concentrations of carbonylated proteins and TBARS (*p* < 0.05) and lowest concentrations of the FRAP antioxidant biomarker (differences were seen in the 2nd DaC tertile, and there was a tendency towards reduction in the 1st tertile, despite statistical signification) after the end of treatment. During this period, it was observed that women in the 3rd tertile showed an increased intake of antioxidants from the polyphenol-rich food and beverages group, compared to women who consumed fewer antioxidants (1st and 2nd tertiles) (Table 3). Women at the lowest tertiles of DaC during treatment showed a significant increase in cabonylated proteins (*p* < 0.001) and TBARS (*p* = 0.037); a decrease in FRAP (*p* = 0.033), after the end of treatment (Table 2); and lower intake of antioxidants from whole cereals, legumes, tubers and roots, total fruits, total vegetables, cruciferous vegetables, citric fruits, and polyphenol-rich foods and beverages during treatment (Table 3).

Multivariate linear regression analysis (Table 4) showed an inverse association between DaC and carbonylated proteins after adjuvant treatment, demonstrated by a 0.204 μmol/L reduction in this biomarker in each 1 mmol of antioxidant daily consumed at T1 (*p* = 0.004).

According to Table 5, multivariate logistic regression analysis showed that lower DaC after adjuvant treatment promotes an 8.06-fold and 0.24-fold increase in the chance of higher lipid hydroperoxide and TBARS concentrations, respectively (*p* = 0.02 and *p* = 0.025), and an 0.12-fold and 0.20-fold increase in the chance of lowered FRAP and GSH levels, respectively (*p* = 0.01 and *p* = 0.044). 

## 4. Discussion

To our knowledge, this is a pioneering investigation regarding the effect of DaC on oxidative stress biomarkers in women undergoing adjuvant treatment for breast cancer. In the present study, women who showed a reduction in DaC after treatment presented significative risk of having higher levels of TBARS and lipid hydroperoxides, and lower levels of GSH and FRAP at the end of adjuvant treatment. A decrease in carbonylated proteins after adjuvant treatment was also observed in response to increased DaC. Important changes in oxidation and antioxidant biomarkers between the beginning and the end of treatment were also observed in different levels of exposure to DaC. Herein, women at the lowest tertiles of DaC, before and during treatment, showed a significant increase in oxidation biomarkers, such as LH and carbonylated proteins, and a decrease in FRAP. It appears that these results indicate that women showing highest DaC before beginning adjuvant treatment and during it exhibit some kind of protection against the aggravation of oxidative stress. This is an expected consequence in women submitted to adjuvant treatment for breast cancer [55,56], since the aim of it is killing breast cancer cells by exacerbating oxidative stress [57,58]. 

In addition to DaC’s positive influence on redox balance, in the present investigation it was also observed that women with breast cancer at the highest level of DaC were also thinner and younger compared to those who were at lower levels of DaC. It is well known that oxidative stress is an important hallmark of the aging process [59], which may contribute to the associations between the lowest tertiles of DaC and oxidative stress observed here. In accordance with the present results, Santos et al. [56] showed that overweight women exhibit lower DaC and antioxidant status biomarkers than women of normal weight diagnosed with breast cancer. Adipose tissue acts as an endocrine organ, producing inflammatory cytokines, which increases the proportion of ROS to antioxidants; in this context, obesity promotes systemic low-grade inflammation and oxidative stress [60]. Moreover, it appears that women at the lowest level of DaC were exposed to higher concentrations of ROS, influenced also by age and BMI. On the other hand, excess weight and aging cannot explain these results in isolation, since multiple linear and logistic regressions confirmed the association between DaC and oxidation and antioxidant biomarkers, in which BMI and age were included as confounders.

Modifications in proteins are mediated by oxidative stress, generating carbonyl groups, which are linked to structural and functional negative changes, including loss of function, alteration in cell cycle, and cancer progression [61,62]. It is well known that women with breast cancer have higher protein carbonyls [63,64,65,66] and lipid hydroperoxides levels than healthy women [67]. In addition, carbonylated proteins are also associated with risk of breast cancer [68], and lipid peroxidation may be inversely associated with survival in women with breast cancer [69]. Contributing to an alteration in redox balance, FRAP levels are decreased in women with breast cancer compared to in healthy women [70], and it seems that chemotherapeutic drugs promote an even greater decline in this biomarker [71], indicating that blood non-enzymatic antioxidant defenses [50] are worsened by the treatment.

Previous investigations have shown that some dietary factors, which are related to non-enzymatic antioxidant defenses, may have an influence on oxidative status in women with breast cancer. Wirth et al. [72] observed that women with a recent history of breast cancer showed lower levels of 8-OH-dG after an intervention with cruciferous vegetables (≥14 cups/week) for 3 weeks. In a Women’s Healthy Eating and Living (WHEL) study, patients previously treated for breast cancer presented a significant decline in 8-Oxo-dG and 8-iso-PGFα levels after a 12-month period of low-fat, high-fruit, and vegetable dietary intervention [73]. Additionally, Yeon et al. [65] found an inverse association between vitamin A and β-carotene intake and 8-OH-dG levels in women with breast cancer. Contributing to the present results, we showed in a recent publication that diet quality score, which includes eating fruits and vegetables according to the Brazilian recommendation for a healthy diet, is positively associated with serum FRAP levels in women diagnosed with breast cancer [55].

Concerning food and food groups which contribute to DaC and may influence oxidation and antioxidant biomarkers, it was observed that the differences in DaC tertiles were attributed only to highest intake of antioxidants from polyphenol-rich foods and beverages, before the beginning of treatment, showing some homogeneity of antioxidant sources in diet at this period. During adjuvant treatment, the differences through tertiles of DaC were linked to higher contribution from whole cereals, legumes, roots and tubers, total fruits, total vegetables, cruciferous vegetables, citric fruits, and polyphenol-rich foods and beverages. Those differences in antioxidant sources by tertiles after treatment may explain why it the strongest associations between the DaC and oxidation and antioxidant biomarkers were observed at the end of the treatment, even though DaC was not significantly different between the periods. Those results indicate that the influence of DaC on attenuating the exacerbation of oxidative stress after adjuvant treatment may be due to diverse antioxidant sources in diet, which may play an important role in synergism of these compounds [74], producing a protective effect.

Despite the positive effect of some dietary factors in oxidative stress triggered by breast cancer, research focused on the associations between dietary antioxidants and oxidative stress in patients undergoing adjuvant treatment is scarce. Oxidative stress is the main cause of chemotherapy and radiotherapy side effects, including nephrotoxicity, cardiotoxicity, hepatoxicity, neurotoxicity, myelosuppression, mucositis, alopecia and gastrointestinal toxicity, which makes the application and effectiveness of those treatments difficult, and so, developing therapeutic strategies is a great of importance for cancer treatment [75]. Antioxidant supplementation during adjuvant treatment is extensively investigated, due to the antioxidants’ power to reduce its side effects [76]. However, this may be harmful for cancer patients, since a reduction in efficacy of treatment through offsetting the apoptotic properties of anticancer drugs is attributed to these kinds of interventions [77]. In the population-based Mamma Carcinoma Risk Factor Investigation (MARIE) with 2223 women, Jung et al. [78] found that concurrent use of antioxidant supplements with adjuvant treatment increased overall mortality and worsened recurrence-free survival. Similar results were found by Lesperance et al. [79] in a cohort study with 90 women, showing that worsened survival was associated with concomitant antioxidant supplementation use with chemotherapy and radiotherapy treatment. Reinforcing the results of these previous investigations, Ambrosone et al. [17] showed in a clinical trial that women who received vitamins A, C, E, carotenoids, and Q-10 coenzime supplementation, before and during chemotherapy for breast cancer, showed an increased risk of recurrence of disease and death. Considering the ambivalent role of the antioxidant effect on patients undergoing cancer treatment, antioxidant supplementation is not recommended for them [80,81]. On the other hand, the controversies concerning antioxidant supplementation go beyond the generalized hypothesis about mechanisms of action. It seems that the same antioxidant molecule may perform both pro-oxidant and antioxidant effect in breast cancer, depending on the concentration, tumor cell type, timing, and environmental conditions [82]. Adverse effects on prognosis in women undergoing adjuvant treatment for breast cancer were already related to antioxidant supplementation, but not to antioxidant intake through food consumption [83]. Further investigations are needed to promote safe, precise, and individual recommendations of antioxidant supplementation for women undergoing adjuvant treatment for breast cancer. At this moment, the National Consensus on Oncologic Nutrition [80] recommends that cancer patients undergoing chemo and radiotherapy should follow a fruit- and vegetable-rich diet (five or more portions daily), and should also avoid using antioxidant supplements which exceed the Dietary Reference Intakes [33], especially among those who are smokers and present alcoholic habit [80].

The present investigation has some limitations. With respect to the dietary intake method of data collection, FFQ is a closed list of foods, and does not allow other components usually consumed to be added, which may lead to the antioxidant capacity of the diet being underestimated. Another point is that it is difficult to be precise about the size of food portions consumed, mainly when the instrument was applied to people with a lower level of understanding. In addition, DaC’s database contains information about foods from a variety of countries, and for some food items, Brazilian data about antioxidant capacity were not available. Another challenge in the calculation of DaC was the unavailability of some food items consumed by Brazilian people in the database. An important limitation to be considered is the heterogeneity of the sample, concerning clinical characteristics as follows: type of breast cancer, timing at diagnosis in respect of menopause status, duration of adjuvant treatment, and number of chemo and radiotherapeutic sessions performed. Those factors may influence oxidative stress and antioxidant biomarkers. 

On the other hand, it is important to consider the strengths of the study. FFQ is an efficient method for estimating food intake because it considers the usual diet, and for this study a validated and adapted version was used, which is based in the sample characteristics. The application of FFQ was performed by trained interviewers, who guided the participants to greater accuracy. DaC was calculated using the most complete database published worldwide, which considers foods from all continents. For those foods that were not available in the database, a specific methodology was used, in which another food item was chosen based on having a similar level of antioxidant content. Concerning the clinical heterogeneity of sample, statistical methods considered this as a potential confounders, which allowed us to clarify the associations between DaC and oxidative stress, reducing the bias.

For instance, the results of the present investigation may be potentially used to improve public health recommendations focused in protecting women undergoing adjuvant treatment for breast cancer, since the main difference is the lower concentrations of antioxidant compounds—which normally are closer to recommended amounts [80]—and the synergy between them [22,23], compared to antioxidant supplementation. Finally, it is possible that the antioxidant intake from foods induces a slight reduction in oxidative stress, with an increase in antioxidant biomarkers and a decrease in oxidation parameters, compared to antioxidant supplementation, promoting a gentle reduction in side effects, without affecting the efficacy of adjuvant treatment for breast cancer. 

## 5. Conclusions

This is the first investigation regarding the impact of DaC on oxidation and antioxidant biomarkers in women undergoing adjuvant treatment. It was shown that higher DaC was associated with lower levels of carbonylated proteins. This association was reinforced when logistic regressions showed that decreased DaC leads to increased chances of having higher oxidation parameters and lower antioxidant biomarkers after adjuvant treatment. The present results also indicate that higher DaC before and during adjuvant treatment promotes a protective effect against exacerbation of oxidative stress provoked by it, and it appears that the variety of antioxidant sources is important to these results, since antioxidant molecules derived from food may act in synergism. Considering the need to develop strategies to promote the slight reduction in oxidative stress in order to reduce side effects, without affecting the efficacy of adjuvant treatment since the concurrent antioxidant supplementation may be harmful, our results may contribute to improving nutritional recommendations for these women. Further investigation may clarify the differences between supplementation and dietary intake of antioxidants, and analyze the direct impact of dietary antioxidant intake on side effects, and the efficacy of adjuvant treatment for breast cancer. 

## Figures and Tables

**Table 1:** Sociodemographic, anthropometric, clinical, and therapeutic characteristics of women with breast cancer according to tertiles of the Dietary antioxidant Capacity (DaC) at T0 (*n* = 70).

		DaC Tertiles at T0		
	1st Tertile	2nd Tertile	3rd Tertile	*p*
Age ^a^ (years)	54.75 (12.0) ^2^	54.91 (8.82) ^3^	47.17 (9.25)	0.016 *
Body Mass Index ^a^ (kg/m^2^)	28.35 (5.54) ^2^	28.41 (3.84) ^3^	25.36 (3.35)	0.030 *
Waist circumference ^a^ (cm)	90.64 (16.1)	92.39 (12.5) ^3^	83.33 (9.53)	0.049 *
Physical Activity Level ^a^	1.34 (0.11)	1.34 (0.10)	1.37 (0.07)	0.447 *
Smoking, *n* (%)				
Yes	3 (12.5)	5 (21.7)	8 (34.8)	0.189 ^#^
No	21 (87.5)	18 (78.3)	15 (65.2)	
Alcohol, *n* (%)				
Yes	0 (0.00)	2 (8.7)	2 (8.7)	0.331 ^#^
No	24 (100)	21 (91.3)	21 (91.3)	
Race, *n* (%)				
White	21 (87.5)	22 (95.7)	23 (100.0)	0.172 ^#^
Brown	3 (12.5)	1 (4.3)	0 (0.0)	
Education				
<8 years	17 (70.8)	17 (73.9)	13 (56.5)	0.755 ^#^
9–11 years	4 (16.7)	3 (13.0)	5 (21.7)	
Estrogen receptor +, *n* (%)				
Yes	13 (59.1)	18 (85.7)	17 (85.0)	0.070 ^#^
No	9 (40.9)	3 (14.3)	3 (15.0)	
Progesterone receptor +, *n* (%)				
Yes	13 (59.1)	17 (80.9)	16 (80.0)	0.189 ^#^
No	9 (40.9)	4 (19.1)	4 (20.0)	
Her2 +, *n* (%)				
Yes	7 (41.2)	0 (00.0)	5 (5.5)	0.099 ^#^
No	10 (58.8)	7 (100)	6 (54.5)	
Triple negative, *n* (%)				
Yes	5 (23.8)	2 (10.0)	0 (00.0)	0.062 ^#^
No	16 (26.2)	18 (90.0)	19 (100.0)	
Tumor classification, *n* (%)				
Invasive carcinoma	21 (87.5)	22 (95.6)	22 (95.6)	0.454 #
Carcinoma in situ	3 (12.5)	1 (4.4)	1 (4.4)	
Tumor stage, *n* (%)				
0	2 (8.3)	0 (0.0)	0 (0.0)	0.524 ^#^
I	8 (33.3)	9 (39.1)	6 (26.1)	
II	9 (37.5)	8 (34.8)	11 (47.8)	
III	5 (20.8)	6 (26.1)	6 (26.1)	
Type of treatment, *n* (%)				
Radiotherapy	5 (20.8)	6 (26.1)	5 (21.7)	0.746 ^#^
Chemotherapy	6 (25.0)	5 (21.7)	9 (39.1)	
Radiotherapy in association with chemotherapy	10 (41.7)	11 (47.8)	8 (34.8)	
No treatment	3 (12.5)	1 (4.35)	1 (4.35)	
Hormone Therapy, *n* (%)				
Tamoxifen	13 (54.2)	14 (60.9)	16 (69.6)	0.313 ^#^
Aromatase inhibitor	1 (4.2)	4 (17.4)	2 (8.7)	
No treatment	10 (41.7)	5 (21.7)	5 (21.7)	
Type of surgery, *n* (%)				
Partial mastectomy	7 (29.2)	5 (21.7)	6 (26.1)	0.914 ^#^
Radical mastectomy	11 (45.8)	12 (52.2)	13 (56.5)	
Sectorectomy	6 (25.0)	6 (26.1)	4 (17.4)	

DaC, Dietary antioxidant Capacity; T0, baseline period; ^a^ mean and standard deviation. ^2^ 1st tertile is different from 3rd tertile. ^3^ 2nd tertile is different from 3rd tertile. * ANOVA trend; ^#^ Chi-square. *p*-value in bold is significant.

**Table 2:** Comparison of changes in the values of oxidative stress biomarkers according to Dietary antioxidant Capacity (DaC) tertiles at T0 and T1 (*n* = 70).

	Biomarker				Biomarker		
	T0	T1	*p*		T0	T1	*p*
DaC Tertile T0	GSH (μmol/L)			DaC Tertile T1	GSH (μmol/L)		
1st tertile ^a^	76.6 (19.5)	77.0 (26.1)	0.958 *	1st tertile ^a^	75.3 (19.9)	76.9 (33.6)	0.850 *
2nd tertile ^a^	75.2 (26.3)	75.3 (31.7)	0.989 *	2nd tertile ^a^	80.0. (22.1)	84.9 (33.3)	0.533 *
3rd tertile ^a^	82.7 (17.5)	85.8 (33.8)	0.670 *	3rd tertile ^a^	78.8 (22.9)	75.5 (23.5)	0.630 *
	FRAP (μmol/L)				FRAP (μmol/L)		
1st tertile ^a^	617.5 (160.1)	528.5 (154.9)	0.070 *	1st tertile ^a^	641.1 (176.5)	554.2 (156.4)	0.033 *
2nd tertile ^a^	683.7 (160.4)	589.8 (177.3)	0.024 *	2nd tertile ^a^	581.6 (148.6)	587.9 (203.9)	0.904 *
3rd tertile ^a^	583.9 (153.9)	607.8 (225.3)	0.682 *	3rd tertile ^a^	667.5 (152.1)	579.4 (203.7)	0.122 *
	Lipid hydroperoxides-log (μmol/L)				Lipid hydroperoxides-log (μmol/L)		
1st tertile ^a^	1.38 (0.73)	1.21 (1.13)	0.520 *	1st tertile ^a^	1.28 (0.70)	1.52 (1.02)	0.392 *
2nd tertile ^a^	1.09 (0.76)	1.43 (1.20)	0.294 *	2nd tertile ^a^	1.24 (0.81)	1.23 (1.09)	0.978 *
3rd tertile ^a^	1.39 (0.75)	1.30 (1.5)	0.773 *	3rd tertile ^a^	1.33 (0.77)	1.17 (1.67)	0.665 *
	Carbonylated proteins-log (μmol/L)				Carbonylated proteins-log (μmol/L)		
1st tertile ^a^	−0.38 (0.35)	−0.04 (0.06)	0.002 *	1st tertile ^a^	−0.33 (0.40)	0.03 (0.23)	<0.001 *
2nd tertile ^a^	−0.35 (0.50)	−0.09 (0.03)	0.019 *	2nd tertile ^a^	−0.21 (0.43)	−0.10 (0.17)	0.300 *
3rd tertile ^a^	−0.04 (0.23)	−0.06 (0.45)	0.888 *	3rd tertile ^a^	−0.24 (0.44)	−0.09 (0.22)	0.220 *
	TBARS-log (μmol/L)				TBARS-log (μmol/L)		
1st tertile ^a^	1.56 (0.41)	2.16 (0.75)	0.006 *	1st tertile ^a^	1.62 (0.47)	2.04 (0.64)	0.037 *
2nd tertile ^a^	1.68 (0.29)	1.98 (0.71)	0.115 *	2nd tertile ^a^	1.64 (0.51)	2.03 (0.80)	0.060 *
3rd tertile ^a^	1.64 (0.61)	1.89 (0.68)	0.170 *	3rd tertile ^a^	1.62 (0.37)	1.97 (0.74)	0.070 *

DaC, Dietary antioxidant capacity; T0, baseline period; T1, period corresponding to post treatment for biomarkers and during treatment for DaC. ^a^ Mean and standard deviation. * paired T-test; FRAP, Ferric reducing antioxidant power; GSH, Reduced glutathione; TBARS, Thiobarbituric acid reactive substances. *p*-value in bold is significant.

**Table 3:** Antioxidant Capacity (aC) from antioxidant-rich food groups according to Dietary antioxidant Capacity (DaC) tertiles at T0 and T1 (*n* = 70).

	T0				T1			
	1st DaC Tertile	2nd DaC Tertile	3rd DaC Tertile	*p*	1st DaC Tertile	2nd DaC Tertile	3rd DaC Tertile	*p*
aC from whole cereals, legumes, tubers and roots (mmol/d) ^a^	0.45 (0.24–0.91)	0.41 (0.30–0.80)	0.63 (0.26–1.01)	**0.736**	0.54 (0.30–0.68)	0.51 (0.26–0.70)	0.92 (0.52–1.75)	0.005 *
aC from Total fruits (mmol/d) ^a^	0.93 (0.67–1.46)	1.22 (0.70–2.06)	1.55 (0.53–2.46)	0.215	1.12 (0.71–1.83)	0.82 (0.44–1.45)	2.06 (1.14–3.02)	0.002 *
aC from Total vegetables (mmol/d) ^a^	0.37 (0.19–0.50)	0.35 (0.19–0.55)	0.39 (0.30–0.60)	0.260	0.38 (0.17–0.54)	0.27 (0.19–0.40)	0.55 (0.30–0.95)	0.040 *
aC from Cruciferous vegetables (mmol/d) ^a^	0.14 (0.05–0.30)	0.10 (0.03–0.30)	0.13 (0.04–0.21)	0.902	0.06 (0.00–0.29)	0.09 (0.04–0.16)	0.18 (0.07–0.35)	0.044 *
aC from Orange and dark green vegetables and fruits (mmol/d) ^a^	0.44 (0.19–0.82)	0.65 (0.33–1.57)	0.75 (0.36–2.15)	0.157	0.34 (0.18–0.66)	0.37 (0.20–0.65)	0.58 (0.26–1.01)	0.141 *
aC from Citric fruits (mmol/d) ^a^	0.23 (0.11–0.56)	0.56 (0.10–1.22)	0.50 (0.18–2.03)	0.186	0.28 (0.13–0.41)	0.22 (0.14–0.37)	0.43 (0.21–0.62)	0.010 *
aC from Red vegetables and fruits (mmol/d) ^a^	0.07 (0.03–0.31)	0.08 (0.03–0.39)	0.08 (0.03–0.49)	0.853	0.13 (0.05–0.43)	0.07 (0.03–0.18)	0.12 (0.04–1.46)	0.255 *
aC from Polyphenol-rich foods and beverages (mmol/d) ^a^	4.21 (2.63–5.05)	7.45 (6.35–8.38)	12.14 (11.2–15.7)	0.0001	3.77 (1.78–4.43)	7.84 (7.44–9.17)	12.0 (9.31–15.2)	0.0001 *

DaC, Dietary antioxidant capacity; aC: antioxidant capacity; T0, baseline period; T1, during adjuvant treatment period; ^a^ Median and interquartile range. * Kruskal–Wallis test. *p*-value in bold is significant.

**Table 4:** Multivariate linear regression analysis of the association between oxidative stress biomarkers and Dietary antioxidant Capacity (DaC) at T0 and T1 (*n* = 70).

Period	Oxidative Stress Biomarkers									
	TBARS-Log *		LH-Log ^†^		Carbonylated Proteins-Log ^‡^		GSH ^§^		FRAP ^II^	
	ß-Adjusted (CI95%)	*p*	ß-Adjusted (CI95%)	*p*	ß-Adjusted (CI95%)	*p*	ß-Adjusted (CI95%)	*p*	ß-Adjusted (CI95%)	*p*
T0	−0.030	0.811	0.134		0.125		0.541		−34.51	
	(−0.283/0.222)		(−0.283/0.552)	0.522	(−0.125/0.375)	0.322	(−10.41/11.49)	0.922	(−111.2/46.17)	0.396
T1	−0.426		−0.144		−0.204		−12.86		−19.20	
	(−1.00/0.153)	0.144	(−1.25/0.961)	0.794	(−0.339/−0.070)	**0.004**	(−32.17/6.44)	0.188	(−130.3/91.92)	0.731

T0, baseline period; T1, post treatment period; CI95%, Confidence interval; DaC, Dietary antioxidant Capacity; FRAP, Ferric reducing antioxidant power; GSH, Reduced glutathione; LH, lipid hydroperoxide; TBARS, Thiobarbituric acid reactive substances. * Adjusted by: age, Body Mass Index (BMI) and alcohol consumption at T0, and by: age, WC (Waist circumference), type of hormone therapy, physical activity level and number of chemotherapeutic sessions at T1. ^†^ Adjusted by: age, WC, alcohol consumption and tumor type at T0, and by: age, Physical Activity Level (PAL), and number of chemotherapeutic sessions at T1. ^‡^ Adjusted by: age and alcohol consumption at T0, and by: age, WC, PAL and tumor type at T1. ^§^ Adjusted by: age, lymph node involvement, PAL and nutritional supplement use at T0, and by: age, race, smoking status and type of treatment at T1. ^II^ Adjusted by: age, WC, alcohol consumption, schooling and nutritional supplement use at T0, and by: age, WC, race, PAL, smoking status, and nutritional supplement use at T1. *p*-value in bold is significant.

**Table 5:** Multivariate logistic regression analysis of the association between changes in Dietary antioxidant Capacity (DaC) and oxidative stress biomarkers between T0 and T1 (*n* = 70).

	Increased Oxidative Stress and Reduced Antioxidant Biomarkers									
	TBARS-Log *		LH-Log ^†^		Carbonylated Proteins-Log ^‡^		GSH ^§^		FRAP ^II^	
	OR (CI95%)	*p*	OR (CI95%)	*p*	OR (CI95%)	*p*	OR (CI95%)	*p*	OR (CI95%)	*p*
Reduced DaC (mmol/day)	0.238 (0.067/0.838)	**0.025**	8.06 (1.39/46.6)	**0.020**	0.497 (0.147/1.680)	0.261	0.200 (0.042/0.956)	**0.044**	0.126 (0.026/0.609)	**0.010**

T0, baseline period; T1, post treatment period; CI95%, Confidence interval; DaC, Dietary antioxidant Capacity; FRAP, Ferric reducing antioxidant power; GSH, Reduced glutathione; LH, lipid hydroperoxide; TBARS, Thiobarbituric acid reactive substances. * Adjusted by: age, Body Mass Index (BMI) and alcohol consumption at T0, and by: age, WC (Waist circumference), type of hormone therapy, physical activity level and number of chemotherapeutic sessions at T1. ^†^ Adjusted by: age, WC, alcohol consumption and tumor type at T0, and by: age, Physical Activity Level (PAL), and number of chemotherapeutic sessions at T1. ^‡^ Adjusted by: age and alcohol consumption at T0, and by: age, WC, PAL and tumor type at T1. ^§^ Adjusted by: age, lymph node involvement, PAL and nutritional supplement use at T0, and by: age, race, smoking status and type of treatment at T1. ^II^ Adjusted by: age, WC, alcohol consumption, schooling and nutritional supplement use at T0, and by: age, WC, race, PAL, smoking status, and nutritional supplement use at T1. *p*-value in bold is significant.

## Supplementary Materials

The following are available online at https://www.mdpi.com/article/10.3390/nu13124324/s1, Table S1: Comparison of the Dietary antioxidant Capacity (DaC), antioxidant Capacity (aC) from food groups and from the major food contributors for DaC, and oxidative stress biomarkers between T0 and T1 (*n* = 70), Table S2: Oxidative stress biomarkers of women with breast cancer according to tertiles of the Dietary antioxidant Capacity (DaC) at T0 and T1 (*n* = 70).
