# Supplementary material for: Dietary Antioxidant Capacity Promotes a Protective Effect against Exacerbated Oxidative Stress in Women Undergoing Adjuvant Treatment for Breast Cancer in a Prospective Study
Source: Nutrients. 2021 Nov 30;13(12):4324. doi: 10.3390/nu13124324 (PMC8707537; doi:10.3390/nu13124324)
Supplement: Supplementary file 1 [file nutrients-13-04324-s001.zip › nutrients-1441123-supplementary.pdf]

Supplementary Material

Table S1. Comparison of the Dietary antioxidant Capacity (DaC), antioxidant Capacity (aC) from food groups and from the major food contributors for DaC, and oxidative stress biomarkers between T0 and T1 (n = 70).

	T0	T1	*p*
DaC[b]	10.7 (8.20-15.3)	11.5 (8.5-15.2)	0.932[*]
aC Whole cereals, legumes, roots and tubers (mmol/d)[b]	0.48 (0.28-0.83)	0.57 (0.39-0.91)	**0.031**[*]
aC from Total fruits (mmol/d)[b]	1.26 (0.70-2.05)	1.18 (0.74-2.23)	0.691[*]
aC from Total vegetables (mmol/d)[b]	0.35 (0.21-0.57)	0.37 (0.19-0.64)	0.849[*]
aC from Cruciferous vegetables (mmol/d)[b]	0.13 (0.04-0.27)	0.12 (0.04-0.25)	0.750[*]
aC from Orange and dark green vegetables and fruits (mmol/d)[b]	0.59 (0.30-1.27)	0.45 (0.24-0.77)	**0.028**[*]
aC from Citric fruits (mmol/d)[b]	0.35 (0.11-1.08)	0.31 (0.16-0.48)	**0.044**[*]
aC from Red vegetables and fruits (mmol/d)[b]	0.09 (0.04-0.41)	0.22 (0.08-0.64)	0.280[*]
aC fom Polyphenol- rich foods and beverages (mmol/d)[b]	7.44 (4.52-11.17)	7.72 (4.05-10.18)	0.602[*]
aC from coffee (mmol/d)[b]	5.58 (3.72-7.44)	5.44 (1.86-7.44)	**0.006**[*]
aC from tangerine (mmol/d)[b]	0.17 (0.01-0.50)	0.01 (0-0.07)	**<0.001**[*]
aC from beans (mmol/d)[b]	0.16 (0.04-0.35)	0.16 (0.06-0.35)	0.210[*]
aC from tea (mmol/d)[b]	0 (0-0.48)	0 (0-1.43)	**<0.001**[*]
aC from mate tea (mmol/d)[b]	0 (0-0.38)	0 (0-0.19)	0.850[*]
aC from guava (mmol/d)[b]	0 (0-0.24)	0 (0-0.24)	0.370[*]
aC from fruit juice (mmol/d)[b]	0 (0-0.22)	0.01 (0-0.05)	0.220[*]
TBARS (μmol/L)[b]	4.81 (4.13-5.68)	8.65 (4.30-13.0)	**<0.001**[*]
Carbonylated proteins (μmol/L)[b]	0.740 (0.570-1.17)	0.940 (0.855-1.07)	**0.020**[*]
Lipid Hydroperoxides (μmol/L)[b]	3.81 (2.84-5.68)	5.59 (1.80-9.75)	**0.050**[*]
GSH (μmol/L)[a]	78.0 (2.60)	79.2 (3.69)	0.800[**]
FRAP (μmol/L)[b]	623.9 (501.1-726.6)	559.2 (447.1-687.1)	**0.030**[*]

DaC, Dietary antioxidant Capacity; aC, antioxidant Capacity; T0, baseline period; T1, period corresponding to post treatment for biomarkers and during treatment for DaC; FRAP, Ferric reducing antioxidant power; GSH, Reduced glutathione; TBARS, Thiobarbituric acid-reactive substances. [a]mean and standard deviation. [b]median and interquartile range. [*]Wilcoxon test. [**]Paired T Test. ***p*-value in bold is significant.**

Table S2. Oxidative stress biomarkers of women with breast cancer according to tertiles of the Dietary antioxidant Capacity (DaC) at T0 and T1 (n = 70).

	CaD Tertiles at T0			*p*	CaD Tertiles at T1			*p*
	1st tertile	2nd tertile	3rd tertile		1st tertile	2nd tertile	3rd tertile	
FRAP (μmol/L)[a]	629.7 (151.4)	661.2 (169.3)	594.8 (160.7)	0.378*	554.2 (156.4)	587.9 (203.9)	579.4 (203.7)	0.821*
GSH (μmol/L)[a]	74.9 (17.7)	78.7 (27.3)	78.8 (18.6)	0.771*	76.9 (33.6)	84.8 (33.3)	75.5 (23.5)	0.539*
TBARS-log (μmol/L)[a]	1.55 (0.381)	1.68 (0.324)	1.63 (0.594)	0.595*	2.04 (0.642)	2.04 (0.810)	1.97 (0.713)	0.948*
LH-log (μmol/L)[a]	1.34 (0.747)	1.16 (0.770)	1.37 (0.731)	0.571*	1.51 (1.01)	1.23 (1.09)	1.17 (1.67)	0.639*
Carbonylated proteins-log(μmol/L)[a]	-0.361 (0.312)	-0.299 (0.522)	-0.068 (0.441)	0.068*	0.036 (0.225)	-0.089 (0.180)	-0.091 (0.225)	0.093*

DaC, Dietary antioxidant Capacity; aC, antioxidant Capacity; T0, baseline period; T1, period corresponding to post treatment for biomarkers and during treatment for DaC; [a]Mean and standard deviation. *ANOVA trend; FRAP, Ferric reducing antioxidant power; GSH, Reduced glutathione; LH, lipid hydroperoxide; TBARS, Thiobarbituric acid reactive substances.
